# Solis Ictus; a Case

**Published:** 1883-01

**Authors:** J. W. Thomson

**Affiliations:** Springfield, Mass.


### SOLIS ICTUS.

J. W. Thomson, M. D., Springfield, Mass.

Called July 15th to see a boy æt. 3 years, in convulsions. The spasms were violent; twitching of hands, arms, and legs with frothing at the mouth during spasmodic action. Face deathly pale, yet felt hot to touch, body burning hot. On opening lids found pupils so much enlarged as to obliterate all appearance of iris. Pulse hardly countable, 180 to 190. Convulsion lasted twenty minutes. Informed that child had had a sour, fetid stool at 6.30 A. M. While in attendance another discharge, involuntary, green, slimy, and of the most unbearable stench.

On inquiry ascertained that he had been playing the day previous, with uncovered head exposed to the rays of the sun. Exhibited Bell.$^{200}$—Dunham's—in water, teaspoonful every ten minutes for an hour, afterward every half hour. Called hurriedly at 2 P. M. There had been another spasm which this time continued ten minutes. It was over when I arrived. Was informed that the little fellow was uncontrollable—slashing round, tearing with his hands, gnashing with his teeth, and kicking with all his might. He would stay proceedings, however, for a moment to drink, and then commence *de novo*. After attack another stool, which was watery, yellow, green and horribly offensive. Pulse, 125. Reaction had evidently commenced. In view of the severity of the attack, continued the Bell. half-hourly. Called at 8.30 P. M. There had been another convulsion at 7 P. M., which, however, only lasted two or three minutes. Another stool, sour, slimy, and yellow. Pulse, 105. Lips, which like the face had been a deathly pale, had now assumed their natural hue. Eyes looked normal. Discontinued medicine.

Called at 10 o'clock next morning. Child had had a good night's rest. There had not been a recurrence of the convulsions. Between 3 and 4 A. M. a stool which was thin, brown, and without slime. Skin moist and normal. Pulse, 90.

I observed the peculiar periodicity of the spams, viz.: at 9, 2, and 7 o'clock, *i. e.*, five hours apart, with the offensive fecal discharge after each attack, yet under the action of the remedy each onset becoming milder. Left a placebo and discharged the case.

Was called again, however, on the following day. The little man had been restless all night, rolling his head from side to side. The pulse was soft, full, and compressible, 108. There had only been one more stool, which I was informed looked natural. Skin was also normal. Gave Sac. Lac. There was no necessity to interfere with the recuperative reaction. I opined that the night's disturbance was simply an effort to throw off the debris of the offending disorder—a settling down to normal activity. To this present writing there has been no return of the malaise. The whole system, brain, heart, and abdominal viscera, had been profoundly disturbed. This was the last throe of the disorderly condition prior to the organism's restoration to its orderly physiological rhythm; like the surging of the billows on the shore after the storm has passed, or scar of remembrance on warrior's face, it was a reverberation, an echo of the storm that had passed.

